# Peripheral telomere length and hippocampal volume in adolescents with major depressive disorder

**DOI:** 10.1038/tp.2015.172

**Published:** 2015-11-10

**Authors:** E Henje Blom, L K M Han, C G Connolly, T C Ho, J Lin, K Z LeWinn, A N Simmons, M D Sacchet, N Mobayed, M E Luna, M Paulus, E S Epel, E H Blackburn, O M Wolkowitz, T T Yang

**Affiliations:** 1 Division of Child and Adolescent Psychiatry, Department of Psychiatry, University of California San Francisco, San Francisco, CA, USA; 2 Department of Clinical Neuroscience, Karolinska Institutet, Stockholm, Sweden; 3 Institute of Interdisciplinary Studies, Amsterdam Brain and Cognition, University of Amsterdam, Amsterdam, The Netherlands; 4 Department of Biochemistry and Biophysics, University of California San Francisco, School of Medicine, San Francisco, CA, USA; 5 Department of Psychiatry, University of California San Diego, La Jolla, CA, USA; 6 The Veterans Affairs Health Care System of San Diego, San Diego, CA, USA; 7 Neuroscience Programs and Department of Psychology, Stanford University, Stanford, CA, USA; 8 Laureate Institute for Brain Research, Tulsa, OK, USA; 9 Department of Psychiatry, University of California San Francisco, School of Medicine, San Francisco, CA, USA

## Abstract

Several studies have reported that adults with major depressive disorder have shorter telomere length and reduced hippocampal volumes. Moreover, studies of adult populations without major depressive disorder suggest a relationship between peripheral telomere length and hippocampal volume. However, the relationship of these findings in adolescents with major depressive disorder has yet to be explored. We examined whether adolescent major depressive disorder is associated with altered peripheral telomere length and hippocampal volume, and whether these measures relate to one another. In 54 unmedicated adolescents (13–18 years) with major depressive disorder and 63 well-matched healthy controls, telomere length was assessed from saliva using quantitative polymerase chain reaction methods, and bilateral hippocampal volumes were measured with magnetic resonance imaging. After adjusting for age and sex (and total brain volume in the hippocampal analysis), adolescents with major depressive disorder exhibited significantly shorter telomere length and significantly smaller right, but not left hippocampal volume. When corrected for age, sex, diagnostic group and total brain volume, telomere length was not significantly associated with left or right hippocampal volume, suggesting that these cellular and neural processes may be mechanistically distinct during adolescence. Our findings suggest that shortening of telomere length and reduction of hippocampal volume are already present in early-onset major depressive disorder and thus unlikely to be only a result of accumulated years of exposure to major depressive disorder.

## Introduction

Major depressive disorder (MDD) and its associated peripheral and central effects is relatively understudied in adolescents, compared with adults, despite the fact that adolescence is a vulnerable period for depression onset.^1^ The prevalence of MDD increases dramatically around puberty,^2^ and the lifetime prevalence of depression in the United States among 13–18-year olds was recently estimated to be 14.3%.^3^ MDD is now considered one of the largest contributors to the United States disease burden in terms of quantified mortality and disability,^4^ and in 2010, depression symptoms were ranked as the second largest contributor worldwide to ‘years lived with disabilities'.^5^ So far, prevention and treatment strategies have not been successful in decreasing the prevalence of adolescent MDD. Potential biomarkers may elucidate risk factors and pathophysiological pathways and aid the development of more targeted and effective preventions and treatments, ideally before the recurrent course of depression is established and associated systemic effects have manifested.

Recently, telomere length (TL), which is considered to be a measure of human cellular aging,^6, 7^ has received considerable attention as a possible biomarker in psychiatric illnesses, offering an explanation for why patients with MDD exhibit an increased risk of developing comorbid and aging-related diseases,^8^ including diabetes,^9^ dementia,^10^ certain types of cancer^11^ and cardiovascular diseases.^12^ Telomeric DNA is comprised of tandem repeat DNA sequences that, together with associated proteins, forms the telomere that caps the chromosome end, providing protection from genome-destabilizing DNA damage responses.^13^ Critical shortening of TL may result in cellular senescence or cell death, and mutations causing insufficient telomere maintenance result in a spectrum of diseases showing overlaps with diseases occurring with population aging.^14^ TL is regarded as a measure of cellular aging in humans as it (a) progressively shortens with every cell division, unless acted upon by the telomere repair enzyme, telomerase;^15^ (b) on average, decreases with advancing age in humans;^6^ and (c) is correlated with current and future physical diseases associated with aging.^7^

Several studies have examined whether accelerated cellular aging is present in depressed adults, yet findings remain inconsistent. Some studies find shorter TL of white blood cells of the peripheral circulation, such as leukocytes or peripheral blood mononuclear cells in MDD,^8, 16, 17^ whereas other studies have not replicated these findings.^18, 19, 20^ Shortening of TL has been reported to be proportional to the total lifetime exposure and duration of MDD, suggesting that accelerated telomere attrition reflects cumulative systemic effects of MDD.^17, 21, 22, 23^ However, another study did not find such a ‘dose-response' relationship^24^ and it was also absent in a late-life cohort study.^25^ Furthermore, TL has been associated with lifestyle factors, for example, poor diet, smoking and decreased physical activity.^26^ To date, it is uncertain whether telomere shortening is the result of chronic depressive illness, lifestyle factors, chromosomal risk factors for developing MDD or a combination of these factors.

In addition to TL shortening, a large body of literature suggests volumetric hippocampal reductions in adult MDD,^27, 28, 29, 30^ but mixed results are also reported.^31, 32^ The hippocampal volume (HV) reduction is especially evident in elderly or chronically-ill samples,^33^ and smaller HV generally seem more apparent in patients suffering from prolonged and/or recurrent episodes of depression.^29, 34^ To date, studies on HV in pediatric MDD show inconsistent findings (for a review see ref. 35), reporting both HV reduction in the MDD samples^36, 37, 38, 39^ and no HV differences between depressed youth and healthy controls (HCs).^40, 41, 42^ However, non-depressed adolescents at high risk for depression also showed smaller HV compared with low-risk participants.^43^ Thus, inconsistencies exist among and between studies in adults and adolescents, and discrepancies of HV differences associated with MDD, remain unresolved.

A series of recent studies has indicated an association between telomere length in both peripheral leukocytes and peripheral blood mononuclear cells and structural brain changes in the hippocampus in populations of adults without MDD^10, 44, 45^ and the only study investigating the relationship between peripheral blood mononuclear cell TL and HV in depressed adults showed no correlation between them.^46^ Whether the telomere shortening and the HV reduction seen in MDD are involved in driving pathophysiological processes of MDD is unclear, nor is it clear whether and how these processes are related. For example, peripheral TL may serve as a proxy for hippocampal TL and hippocampal TL shortening may be the underlying mechanism by which HV is reduced in MDD. As telomere shortening (in mice) may retard neuritogenesis even in adult mouse brain tissue,^47^ and as newly generated neurons are hypersensitive to telomere damage,^48^ we hypothesize that a potential shortening of TL in hippocampal neurons may disrupt or dampen plasticity and, over time, cause a reduction of HV. Alternatively, both shortened peripheral TL and reduced HV may be the result of a common mediating factor, such as oxidative stress.^49, 50^ Many factors may thus potentially influence both the TL and HV in depressed populations, such as duration of illness, age of onset, number of recurrent episodes, ongoing or previous medication, psychiatric comorbidity and lifestyle factors.^51^ Studying early onset of MDD allows most of these potential confounders to be minimized. Investigations of the association between peripheral TL and changes of HV, especially early in the disease progression, may help further elucidate the pathophysiological pathways of early MDD and could potentially identify new treatment targets.

In this study, TL of saliva sample cells and HV are compared between adolescent MDD and HC with the following aims: (a) to investigate whether depressed unmedicated adolescents show shorter salivary TL as compared with well-matched HC, (b) to determine whether adolescent MDD is associated with reduced HV relative to HC and (c) to examine the relationship between peripheral TL and HV in adolescent MDD. The question of whether there is an association between TL and HV in MDD has never been examined in adolescents, and based on studies of adults there is rationale for expecting both a positive association between TL and HV as well as no such association. One previous study on adult MDD^46^ showed that shorter TL was not related to reduced HV, but such a relationship has been shown in other types of samples.^10, 44, 45^

## Materials and methods

### Participants

A total sample of 117 participants (age range 13–18 years) was selected from a larger ongoing study at the University of California, San Diego.^52, 53^ HC adolescents were recruited from the San Diego area through posted flyers, email and the Internet. MDD adolescents were recruited from 35 adolescent psychiatric clinics dispersed through the San Diego County area. After application of the exclusion criteria (see Supplementary Information), the data from 54 adolescents with a Diagnostic and Statistical Manual of Mental Disorders, fourth edition diagnosis of MDD established by the Schedule for Affective Disorders and Schizophrenia for School-Age Children—Present and Lifetime Version^54^ and 63 well-matched HCs were used in the statistical analyses. In all the participants, depression severity was clinically assessed with the Children's Depression Rating Scale-Revised (CDRS-R)^55^ and self-assessment scales (see Supplementary Information) at the time of magnetic resonance imaging (MRI) scanning. Saliva samples were also collected at the time of MRI scanning. Early adversity was assessed by retroactive self-report using the Childhood Trauma Questionnaire,^56^ and collected later in the study, to test the hypothesis that childhood trauma may be related to TL shortening, HV reduction and adolescent depression.^57, 58^ For a complete description of the assessments, see Supplementary Information. The institutional review boards of the University of California, San Diego and University of California, San Francisco, Rady Children's Hospital and the County of San Diego approved this study. Participants and their parents/legal guardians provided written informed assent and consent, respectively. Participants received monetary compensation for their time.

### Telomere length measurement

Genomic DNA was purified from 500 μl of saliva collected in an Oragene DNA kit (DNA Genotek, Kanata, Ontario, Canada) with the DNA Agencourt DNAdvance kit (cat #A48705, Beckman Coulter Genomics, Brea, CA, USA) according to the instruction of the manufacturer. DNA was quantified by Quant-iT PicoGreen dsDNA Assay Kit (cat #P7589, Life Technologies, Grand Island, NY, USA) and run on 0.8% agarose gels for integrity check. Degraded DNA samples were excluded from TL analysis. The quantitative PCR TL measurement assay was adapted from the original method,^59^ and was performed by the Blackburn Lab at the Department of Biochemistry and Biophysics at University of California, San Francisco, and is described in detail elsewhere.^60^ The personnel who performed the assay received de-identified samples and were blind to all other measurements. For a more detailed description of the procedure, see Supplementary Information.

### Image data acquisition and preprocessing

All the MRI data were acquired on a 3 T MR750 GE scanner (GE Healthcare, Milwaukee, WI, USA) at the University of California, San Diego Center for functional MRI. A fast-spoiled gradient-recalled echo sequence was used to collect T1-weighted images: TR=8.1 ms, TE=3.17ms, TI=450ms, flip angle=12°, 256 × 256 matrix, FOV=250 × 250 mm, 168 sagittal slices with a voxel size of 0.98 × 0.98 × 1 mm. Data were preprocessed and analyzed with AFNI^61^ and FSL.^62^ AFNI^61^ was only used to remove non-brain tissue. The resultant images were segmented into gray and white matter, and cerebrospinal fluid using FAST.^63^ Total brain volume (TBV) was calculated from the gray and white matter segments. Bilateral HVs were estimated with FSL-FIRST.^64^ FSL version 5.0.8 was used to analyze all the participants, and all the segmentations were visually inspected.

### Statistical analysis

Statistical analyses were performed using R.^65^ Between-group comparisons of the demographic variables were conducted by Welch *t*-tests, *χ*^2^ tests and Wilcoxon rank-sum tests. To investigate between-group differences in TL, we conducted an analysis of covariance with diagnostic group (MDD, HC) as a between-subjects factor and sex and age (in years) as within-subjects factors. Analysis of HV (left and right separately) was conducted similarly, insofar as we used analyses of covariance with group as between-subjects and age and sex as within-subject factors. TBV was also included in these models to account for inter-individual differences in brain volume.^66^ Examination of TBV between groups was performed as for the TL analysis with an additional model investigating the interaction of group and age on TBV. To investigate the association of HV and peripheral TL, a linear regression model was computed with HV (left, right separately) as the dependent variable and TL, age, sex, diagnostic group (for across group analysis), and TBV as predictors. Finally, linear regressions were conducted to examine associations between demographic (age) and clinical characteristics (CDRS-R, CTQ) on both TL and HV. This was performed separately within MDDs and HCs and HV regressions were controlled for TBV. Analyses of covariance and linear regressions for TL and TBV were considered significant at *P*<0.05. The analyses of HVs were Bonferroni corrected for two hemispheres and were consequently considered significant at *P*<0.025. Personnel performing these analyses were not blind to participant grouping.

## Results

### Sample characteristics

The mean age of our total sample was 15.8 years (s.d.=1.32 years, range 13.1–18.1 years), consisting of 54.7% of females. The groups did not differ significantly in age, sex, pubertal status and socioeconomic status (all *P*>0.05), and no left-handed persons were included in the sample. For a detailed description of these assessments, see Supplementary Information Comorbid psychiatric disorders were assessed during the KSAD-PL interview, a diagnostic interview the MDD participants had to undergo to confirm a primary diagnosis of MDD, and, therefore, eligibility for the current study. For a detailed description of current comorbidities in our MDD sample, see Table 1. To investigate the effect of the number of comorbidities on TL and HV, we conducted analyses of variance with the number of comorbidities (0, 1, 2+) as a between-subjects factor (and total brain volume as a within-subject factor in case of the hippocampal volume analyses), but found no effect of the number of comorbidities on either TL or HV in the MDDs (all *P*>0.4). See Supplementary Information for more details. As anticipated, MDD subjects reported higher levels of depression (CDRS-R) and childhood trauma (CTQ) than the HCs (both *P*<0.001; see Table 1).

### Telomere length

**Figure 1:**
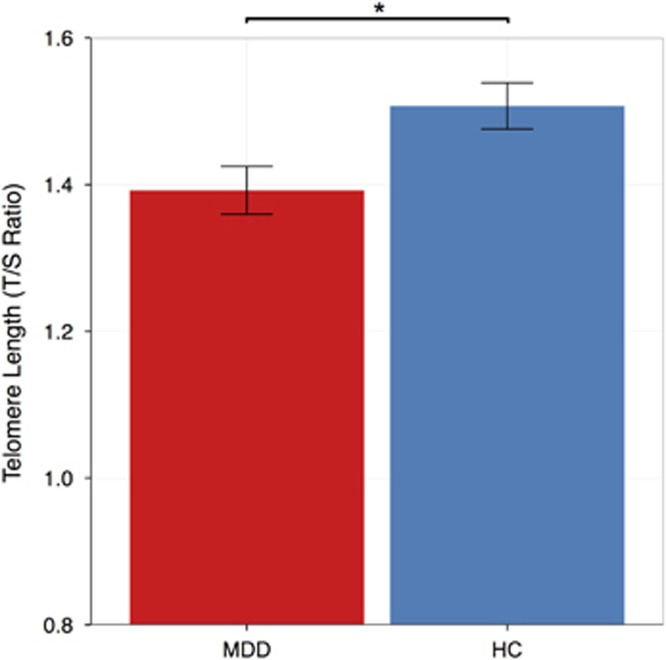
Telomere length (T/S ratio, mean±s.e.m.) in adolescents with major depressive disorder (MDD) and healthy controls (HCs). Adolescents with MDD exhibited significantly shorter age- and sex-adjusted telomere length than HCs, F_(1,113)_=6.59, *P=0.01.

Significantly shorter salivary TL was observed in the depressed subjects compared with HCs (mean T/S ratio±s.d. MDD: 1.39±0.24, HC: 1.51±0.25; F_(1,113)_= 6.59, *P*<0.05). There was also a main effect of age (F_(1,113)_=4.85*, P*<0.05), with older subjects showing shorter TL. Mean TL of both groups is presented in Figure 1. TL was not significantly associated with CDRS-R scores or CTQ total score in either group (all *P*>0.19).

### Hippocampal volume

**Figure 2:**
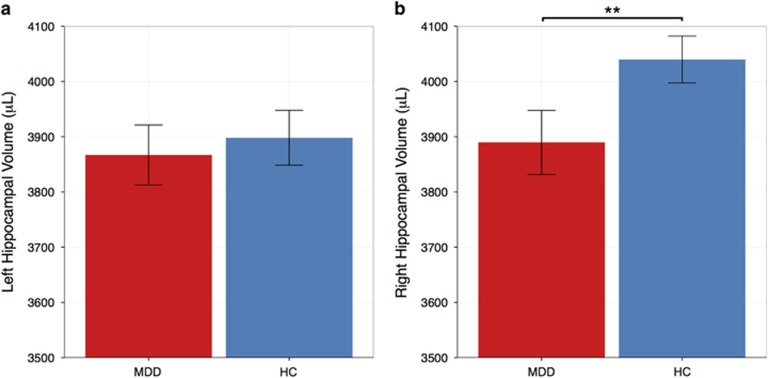
Left and right hippocampal volumes (mean±s.e.m.) adjusted for age, sex and total brain volume in adolescents with major depressive disorder (MDD) and healthy controls (HCs). Although there was no significant group difference observed for the left hippocampus (**a**), the depressed group exhibited significantly smaller right hippocampal volumes, F_1,112_=6.91, **P=0.009 (**b**).

MDD subjects had significantly smaller right HVs than HCs after adjusting for age, sex and TBV (mean HV±s.d. MDD: 3890 μl±426, HC: 4040 μl±336; F_(1,112)_= 6.91, *P<*0.01), but no difference in the left HV was observed (F_(1,112)_= 0.23, *P=*0.63); see Figure 2. A main effect of sex was seen for both left and right HV (both *P<*0.001), with males showing larger right and left hippocampi than females. TBV was lower in adolescents with MDD compared with HCs (mean TBV±SD MDD: 1 074 655 μl±111 958, HC: 1 142 252 μl±96 964; F_(1,113)_=19.7, *P<*0.001), after controlling for age and sex. However, an additional model showed a significant interaction between group and age on TBV (F_(1,112)_=4.22, *P<*0.05), after controlling for sex. HV (left, right) was not significantly associated with CDRS-R (both *P>*0.033) or CTQ total score (both *P*>0.36) in either group.

### Relationship between HV and TL

We examined the association between HV (left and right separately) and TL across groups and found that TL did not significantly predict the left (*β*=−24.4, *t*_(111)_=−0.18, *P*>0.86) or right HV (*β*=53, *t*_(111)_=−0.44, *P*>0.66), when correcting for age, sex, diagnostic group and TBV. Separate within-group analyses did not detect any significant relationship between HV and TL (all *P*>0.08). Additional models not adjusting for covariates also did not reveal any significant associations between TL and HV (see Supplementary Information).

## Discussion

To our knowledge, this is the first study to examine peripheral TL and HV in adolescent depression. The study yielded three main findings: first, peripheral TL was significantly shorter in adolescents with MDD compared with HCs. Second, MDD subjects had reduced right, but not left, HV compared with HC. Finally, no significant associations between TL and HV, within or across groups, were found.

The first finding provides evidence that shorter salivary TL is present in a sample of adolescents diagnosed with MDD as compared with HC. As the depressed adolescents were not receiving any psychotropic medication, our observation of TL shortening, in the MDD group, suggests that shortened telomeres may be a marker that is associated with adolescent onset MDD and not solely result from extended MDD exposure or the cumulative effect of MDD, as previously suggested in adult samples.^22, 23^ Importantly, even though the adolescents may have had limited exposure to lifestyle factors associated with cellular aging as compared with older samples, the shorter TL in our depressed sample may still be related to lifestyle or other life factors. The lack of an association between depression severity scores and TL in our sample also supports the notion that TL shortening may either be a part of depression etiology, or else reflective of other damaging factors that are part of its etiology or both. Interestingly, non-depressed girls with depressed mothers, that is, girls at high risk for developing MDD also show shorter salivary TL compared with age-matched low-risk girls.^60^ These findings could be explained by a possible genetic effect on TL and/or increased exposure to factors that could influence TL even before the onset of MDD, such as maternal stress during pregnancy^67^ and early-life stress in the MDD group.^60^ The present study, however, found no significant correlations between either TL or HV and childhood trauma.

The second finding of decreased right, although not left, HV in adolescent MDD is consistent with the consensus of reduced HV in adult MDD^27, 28, 29^ and what is generally reported in adolescent MDD, (for a review, see ref. 35). Analogous to our TL results, this suggests that the hippocampus may be negatively affected early in the course of MDD. Furthermore, HV volume changes may also be involved in the etiology of the disorder and not only after extended duration and/or recurrent episodes of depression as has previously^27, 29, 68, 69^ but not consistently^31, 70^ been reported. Consistent with our results, some studies show HV reductions in adolescents at risk of developing MDD even before clinical manifestation of the disease.^37, 38^ Recent findings show associations between hippocampal volume abnormalities and several mechanisms involved in stress-related disorders and MDD, such as the messenger RNA expression of glucocorticoid inducible genes,^71^ glucocorticoid receptor methylation,^72^ genetic polymorphisms associated with variation in pro-inflammatory cytokine levels^73^ and increased oxidative stress.^49^

The asymmetrical right-sided HV reduction found in our MDD sample was present independent of age and sex. Previous literature on adolescents at risk for MDD and adults with MDD report inconsistent findings with regard to the lateralization of hippocampal volume reductions with both left-sided,^36^ right-sided^36^ and bilateral volume reductions.^37, 38^ Furthermore, hippocampus asymmetries are also prevalent in the normal population independent of MDD and the neurodevelopmental processes; other possible mechanisms and clinical implications of these asymmetries are unknown.^74^ More research into the causes and implications of lateralization of structural changes in the hippocampus is warranted in general and in MDD in particular.

The third finding showed a lack of an association between TL and HV, replicating the one similar study performed in adults. This may suggest that these cellular and neural features may be mechanistically distinct, a least in this age group. The relationship between peripheral TL and HV has only been previously investigated in one small study with an MDD population wherein leukocyte TL was found to be unrelated to HV in both MDD and HC.^46^ Interestingly, TL has been linked to variance in HV in the general population,^75^ in early-stage dementia^10^ and in adolescents with early-childhood stress.^76^ In addition, experimental studies have shown that oxidative stress is associated with telomere shortness in the periphery, and associated with reduced neurogenesis and increased neuronal death in the hippocampus.^77^ Several, but not all, studies of MDD report high levels of peripheral markers of oxidative stress, (for a review, see ref. 78). Peripheral blood circulation cell telomerase activity, rather than TL, was recently found to be positively correlated with HV in adult MDD^79^ but not in HC, suggesting that decreased telomerase activity, which is a cellular enzyme compensating for the loss of telomeric repeats and preserving TL^13, 20^ may thus be more directly related to HV reduction than TL itself, at least regarding peripheral TL and telomerase measures.

Our relatively large sample size lends confidence to our findings and a major strength of this study is the reduction of confounding factors on TL and HV, such as psychotropic medications. Nevertheless, our study is not without limitations. First, the relatively young age range of our MDD sample limited the possible duration of MDD as compared with adult samples, but unfortunately, we lack more precise data on the age of onset or the duration of depression in our samples. Second, the design was cross-sectional in nature, yet the cellular and neural outcomes associated with MDD are likely to be influenced by neurodevelopment. Third, telomerase cannot be analyzed from salivary samples; therefore, the previously described relationship between telomerase activity in peripheral blood mononuclear cells such as leukocytes and HV could not be validated. Saliva contains both epithelial cells and leukocytes, which are not possible to separate in our analysis but known to be highly correlated, suggesting systematic effects of TL regulation.^80^ Therefore, the use of saliva as the source of DNA for TL measurement is justified. TL in human tissues generally shows significant correlations within individuals,^81^ even though human postmortem investigations shows no strong correlations between TL in peripheral leukocytes and other tissues including brain tissue.^82^ It is not fully known how the TL in peripheral cells relates to TL in the hippocampal neurons. However, shorter hippocampal telomere length has been found in well-defined depression models of rats.^83^

Certain limitations arise from our choice of FSL-FIRST to perform subcortical segmentation. Although Freesurfer^84^ estimates of HV have been shown to have higher correlation with the volumes derived from manual segmentations,^85^ Freesurfer tends to overestimate HV in younger age groups compared with manual segmentations.^86^ In addition, although the data set used to train FSL-FIRST to perform automated segmentation consisted of a large sample (*n*=336) with a wide age range (4.2–87 years) of controls and participants with different pathophysiologies,^64^ we cannot rule out the presence of a systematic bias attributable to the mismatch between the sample used in the present study and that used to train FSL-FIRST.

Finally, the original study was not designed to assess early adversity and the CTQ was therefore added later in the study. Consequently, CTQ data were not available from the full samples (see Table 1), which reduced our power to detect associations. Although significantly higher CTQ scores were found in the MDD sample compared with the HC, no associations were found between CTQ-score and TL or HV within the MDD sample. Our finding does not support the hypothesis that TL and HV alterations observed in adolescent MDD are explained by childhood trauma alone, but rather suggests that MDD may influence these outcomes.

To fully capture how TL and HV changes relate to MDD pathophysiology and disease progression, future studies with prospective longitudinal designs are needed, ideally combining MRI with assessments of clinical data including adverse childhood experiences, putative biochemical mediators of TL shortening and/or HV diminution, for example, oxidative stress, systemic inflammation and telomerase activity.

In conclusion, this is the first study to reveal shorter salivary TL in adolescent MDD, despite their relatively short illness history and limited exposure to lifestyle factors associated with cellular aging. This novel finding supports the notion that telomere shortening is not only associated with chronic depressive illness, but present early in the course of MDD, possibly representing a genetic and/or acquired risk factor for MDD development. We also show that the right HV is reduced in early-onset MDD. This suggests that HV reduction is present before MDD or that the hippocampus may be negatively affected early in disease progression and not only after recurrent and/or elongated periods of depression. The absence of a significant relationship between peripheral TL and HV in our sample suggests that these cellular and neural outcomes associated with MDD may be mechanistically distinct, at least during adolescence. Given that this association may be different in other age groups, further exploration is warranted.

## Figures and Tables

**Table 1:** Participant characteristics

*Characteristic*	*MDD*^a^	*HC*^a^	*Statistic*^b^	P-*value*	*Significance*
Number of participants (*n*)	54	63	*χ*^2^ (1.00)=0.55	0.46	
Gender (M/F)	19/35	34/29	*χ*^2^ (1.00)=3.42	0.06	
Age at time of scan (years)	15.9±0.2 (13.1–18.1)	15.8±0.2 (13.1–17.9)	*t* (107.83)=0.49	0.62	
Hollingshead Socioeconomic Score	40±38.5 (11–70)^†^	29±27 (0–69) [1]^†^	*W*=1938	0.14	
Tanner Score	4±0.5 (3–5)^†^	4±0.8 (3–5)^†^	*W*=1885	0.29	
Wechsler Abbreviated Scale of Intelligence (full)	100.4±1.7 (77–129)	107.5±1.5 (83–137)	*t* (109.61)=−3.20	<0.01	**
Wechsler Abbreviated Scale of Intelligence (performance)	98.3±1.7 (62–122)	105.7±1.5 (84–126)	*t* (111.10)=−3.31	<0.01	**
Wechsler Abbreviated Scale of Intelligence (verbal)	102.3±2 (72–132)	108±1.5 (77–141)	*t* (101.04)=−2.26	0.026	*
Children's Global Assessment Scale	65±18.8 (41–85)^†^	90±10 (70–100)^†^	*W*=34	<0.001	***
Children's Depression Rating Scale (standardized)	71.1±1.2 (55–85)	32.6±0.4 (30–44)	*t* (68.24)=30.44	<0.001	***
Beck Depression Inventory II	26.7±1.5 (4–47)	2.7±0.4 (0–15) [1]	*t* (61.91)=15.65	<0.001	***
Children's Depression Inventory	24.1±1.1 (6–38)	5±0.4 (2–15) [1]	*t* (66.14)=15.77	<0.001	***
Multidimensional Anxiety Scale for Children (standardized)	58.4±1.4 (34–83) [4]	42.4±1.1 (26–61) [5]	*t* (98.03)=8.97	<0.001	***
CTQ (total)	64.9±3.2 (41–94)	42.4±0.9 (37–53)	*t* (24.44)=6.82	<0.001	***
Number of participants in CTQ analysis	22	25	*χ*^2^ (1.00)=0.09	0.77	

*Current Comorbid DSM-IV Diagnoses*					
No comorbid diagnosesc,^d^	20				
1 comorbid diagnosis^d^	17				
2 comorbid diagnoses^d^	12				
>2 comorbid diagnoses^d^	1				
					
Generalized anxiety disorder	16				
Social anxiety disorder	2				
Panic disorder	1				
Specific phobia	4				
Posttraumatic stress disorder	5				
Adjustment disorder	1				
Attention deficit hyperactivity disorder	8				
Alcohol/substance dependence	1				
Conduct disorder	2				
Oppositional defiance disorder	3				
Eating disorder (not otherwise specified)	2				

Abbreviations: CTQ, Childhood Trauma Questionnaire; DSM-IV, Diagnostic and Statistical Manual of Mental Disorders, fourth edition; F, female; HC, healthy control; IQR, interquartile range; M, male; MDD, major depressive disorder; *P<0.05; **P<0.01; ***P<0.001.

a Mean±s.e.m. (min–max) or median±IQR (min–max) if indicated by ^†^. The optional number in [ ] indicates the number of missing data items.

b Statistic: W, Wilcox rank-sum test; *χ*^2^, *χ*^2^ test for equality of proportions; *t*, Student's *t*-test.

c Refers to the absence of current DSM-IV diagnoses listed in this table.

d The KSADS-PL of four MDD subjects were mislaid after the initial diagnosis. Consequently, no comorbidity information is available on those subjects.

## References

[bib1] Angold A, Costello EJ, Worthman CM. Puberty and depression: the roles of age, pubertal status and pubertal timing. Psychol Med 1998; 28: 51–61. 9483683 10.1017/s003329179700593x

[bib2] Hankin BL, Abramson LY, Moffitt TE, Silva PA, McGee R, Angell KE. Development of depression from preadolescence to young adulthood: emerging gender differences in a 10-year longitudinal study. J Abnorm Psychol 1998; 107: 128–140. 9505045 10.1037//0021-843x.107.1.128

[bib3] Avenevoli S, Swendsen J, He JP, Burstein M, Merikangas KR. Major depression in the national comorbidity survey-adolescent supplement: prevalence, correlates, and treatment. J Am Acad Child Adolesc Psychiatry 2015; 54: 37–44. 25524788 10.1016/j.jaac.2014.10.010 PMC4408277

[bib4] Ferrari AJ, Charlson FJ, Norman RE, Patten SB, Freedman G, Murray CJ et al. Burden of depressive disorders by country, sex, age, and year: findings from the Global Burden of Disease Study 2010. PLoS Med 2013; 10: e1001547. 24223526 10.1371/journal.pmed.1001547 PMC3818162

[bib5] Vos T, Flaxman AD, Naghavi M, Lozano R, Michaud C, Ezzati M et al. Years lived with disability (YLDs) for 1160 sequelae of 289 diseases and injuries 1990-2010: a systematic analysis for the Global Burden of Disease Study 2010. Lancet 2012; 380: 2163–2196. 23245607 10.1016/S0140-6736(12)61729-2 PMC6350784

[bib6] Blackburn EH. Telomere states and cell fates. Nature 2000; 408: 53–56. 11081503 10.1038/35040500

[bib7] Wolkowitz OM, Reus VI, Mellon SH. Of sound mind and body: depression, disease, and accelerated aging. Dialogues Clin Neurosci 2011; 13: 25–39. 21485744 10.31887/DCNS.2011.13.1/owolkowitz PMC3181963

[bib8] Wolkowitz OM, Epel ES, Reus VI, Mellon SH. Depression gets old fast: do stress and depression accelerate cell aging? Depress Anxiety 2010; 27: 327–338. 20376837 10.1002/da.20686

[bib9] McIntyre RS, Soczynska JK, Konarski JZ, Woldeyohannes HO, Law CW, Miranda A et al. Should depressive syndromes be reclassified as "Metabolic Syndrome Type II"? Ann Clin Psychiatry 2007; 19: 257–264. 18058283 10.1080/10401230701653377

[bib10] Grodstein F, van Oijen M, Irizarry MC, Rosas HD, Hyman BT, Growdon JH et al. Shorter telomeres may mark early risk of dementia: preliminary analysis of 62 participants from the nurses' health study. PLoS One 2008; 3: e1590. 18795148 10.1371/journal.pone.0001590 PMC2536511

[bib11] Willeit P, Willeit J, Mayr A, Weger S, Oberhollenzer F, Brandstatter A et al. Telomere length and risk of incident cancer and cancer mortality. JAMA 2010; 304: 69–75. 20606151 10.1001/jama.2010.897

[bib12] Epel ES, Lin J, Wilhelm FH, Wolkowitz OM, Cawthon R, Adler NE et al. Cell aging in relation to stress arousal and cardiovascular disease risk factors. Psychoneuroendocrinology 2006; 31: 277–287. 16298085 10.1016/j.psyneuen.2005.08.011

[bib13] Blackburn EH. Switching and signaling at the telomere. Cell 2001; 106: 661–673. 11572773 10.1016/s0092-8674(01)00492-5

[bib14] Armanios M, Blackburn EH. The telomere syndromes. Nat Rev Genet 2012; 13: 693–704. 22965356 10.1038/nrg3246 PMC3548426

[bib15] Blackburn EH. Structure and function of telomeres. Nature 1991; 350: 569–573. 1708110 10.1038/350569a0

[bib16] Garcia-Rizo C, Fernandez-Egea E, Miller BJ, Oliveira C, Justicia A, Griffith JK et al. Abnormal glucose tolerance, white blood cell count, and telomere length in newly diagnosed, antidepressant-naive patients with depression. Brain Behav Immun 2013; 28: 49–53. 23207109 10.1016/j.bbi.2012.11.009 PMC3587123

[bib17] Simon NM, Smoller JW, McNamara KL, Maser RS, Zalta AK, Pollack MH et al. Telomere shortening and mood disorders: preliminary support for a chronic stress model of accelerated aging. Biol Psychiatry 2006; 60: 432–435. 16581033 10.1016/j.biopsych.2006.02.004

[bib18] Shaffer JA, Epel E, Kang MS, Ye S, Schwartz JE, Davidson KW et al. Depressive symptoms are not associated with leukocyte telomere length: findings from the Nova Scotia Health Survey (NSHS95), a population-based study. PLoS One 2012; 7: e48318. 23133583 10.1371/journal.pone.0048318 PMC3485011

[bib19] Hoen PW, Rosmalen JG, Schoevers RA, Huzen J, van der Harst P, de Jonge P. Association between anxiety but not depressive disorders and leukocyte telomere length after 2 years of follow-up in a population-based sample. Psychol Med 2013; 43: 689–697. 22877856 10.1017/S0033291712001766

[bib20] Needham BL, Mezuk B, Bareis N, Lin J, Blackburn EH, Epel ES. Depression, anxiety and telomere length in young adults: evidence from the National Health and Nutrition Examination Survey. Mol Psychiatry 2014; 20: 520–528. 25178165 10.1038/mp.2014.89 PMC4346549

[bib21] Wikgren M, Maripuu M, Karlsson T, Nordfjall K, Bergdahl J, Hultdin J et al. Short telomeres in depression and the general population are associated with a hypocortisolemic state. Biol Psychiatry 2012; 71: 294–300. 22055018 10.1016/j.biopsych.2011.09.015

[bib22] Wolkowitz OM, Mellon SH, Epel ES, Lin J, Dhabhar FS, Su Y et al. Leukocyte telomere length in major depression: correlations with chronicity, inflammation and oxidative stress—preliminary findings. PLoS One 2011; 6: e17837. 21448457 10.1371/journal.pone.0017837 PMC3063175

[bib23] Verhoeven JE, Revesz D, Epel ES, Lin J, Wolkowitz OM, Penninx BW. Major depressive disorder and accelerated cellular aging: results from a large psychiatric cohort study. Mol Psychiatry 2014; 19: 895–901. 24217256 10.1038/mp.2013.151

[bib24] Hartmann N, Boehner M, Groenen F, Kalb R. Telomere length of patients with major depression is shortened but independent from therapy and severity of the disease. Depress Anxiety 2010; 27: 1111–1116. 21053332 10.1002/da.20749

[bib25] Schaakxs R, Verhoeven JE, Oude Voshaar RC, Comijs HC, Penninx BW. Leukocyte telomere length and late-life depression. Am J Geriatr Psychiatry 2015; 23: 423–432. 25028345 10.1016/j.jagp.2014.06.003

[bib26] Lin J, Epel E, Blackburn E. Telomeres and lifestyle factors: roles in cellular aging. Mutat Res 2012; 730: 85–89. 21878343 10.1016/j.mrfmmm.2011.08.003

[bib27] Bremner JD, Narayan M, Anderson ER, Staib LH, Miller HL, Charney DS. Hippocampal volume reduction in major depression. Am J Psychiatry 2000; 157: 115–118. 10618023 10.1176/ajp.157.1.115

[bib28] Frodl T, Meisenzahl EM, Zetzsche T, Born C, Groll C, Jager M et al. Hippocampal changes in patients with a first episode of major depression. Am J Psychiatry 2002; 159: 1112–1118. 12091188 10.1176/appi.ajp.159.7.1112

[bib29] MacQueen GM, Campbell S, McEwen BS, Macdonald K, Amano S, Joffe RT et al. Course of illness, hippocampal function, and hippocampal volume in major depression. Proc Natl Acad Sci USA 2003; 100: 1387–1392. 12552118 10.1073/pnas.0337481100 PMC298782

[bib30] Cole J, Costafreda SG, McGuffin P, Fu CH. Hippocampal atrophy in first episode depression: a meta-analysis of magnetic resonance imaging studies. J Affect Disord 2011; 134: 483–487. 21745692 10.1016/j.jad.2011.05.057

[bib31] McKinnon MC, Yucel K, Nazarov A, MacQueen GM. A meta-analysis examining clinical predictors of hippocampal volume in patients with major depressive disorder. J Psychiatry Neurosci 2009; 34: 41–54. 19125212 PMC2612082

[bib32] Treadway MT, Waskom ML, Dillon DG, Holmes AJ, Park MT, Chakravarty MM et al. Illness progression, recent stress, and morphometry of hippocampal subfields and medial prefrontal cortex in major depression. Biol Psychiatry 2015; 77: 285–294. 25109665 10.1016/j.biopsych.2014.06.018 PMC4277904

[bib33] Savitz JB, Drevets WC. Imaging phenotypes of major depressive disorder: genetic correlates. Neuroscience 2009; 164: 300–330. 19358877 10.1016/j.neuroscience.2009.03.082 PMC2760612

[bib34] Lorenzetti V, Allen NB, Fornito A, Yucel M. Structural brain abnormalities in major depressive disorder: a selective review of recent MRI studies. J Affect Disord 2009; 117: 1–17. 19237202 10.1016/j.jad.2008.11.021

[bib35] Hulvershorn LA, Cullen K, Anand A. Toward dysfunctional connectivity: a review of neuroimaging findings in pediatric major depressive disorder. Brain Imaging Behav 2011; 5: 307–328. 21901425 10.1007/s11682-011-9134-3 PMC3216118

[bib36] MacMaster FP, Kusumakar V. Hippocampal volume in early onset depression. BMC Med 2004; 2: 2. 14969587 10.1186/1741-7015-2-2 PMC333436

[bib37] MacMaster FP, Mirza Y, Szeszko PR, Kmiecik LE, Easter PC, Taormina SP et al. Amygdala and hippocampal volumes in familial early onset major depressive disorder. Biol Psychiatry 2008; 63: 385–390. 17640621 10.1016/j.biopsych.2007.05.005 PMC2268763

[bib38] Rao U, Chen LA, Bidesi AS, Shad MU, Thomas MA, Hammen CL. Hippocampal changes associated with early-life adversity and vulnerability to depression. Biol Psychiatry 2010; 67: 357–364. 20015483 10.1016/j.biopsych.2009.10.017 PMC2821020

[bib39] Caetano SC, Fonseca M, Hatch JP, Olvera RL, Nicoletti M, Hunter K et al. Medial temporal lobe abnormalities in pediatric unipolar depression. Neurosci Lett 2007; 427: 142–147. 17949901 10.1016/j.neulet.2007.06.014

[bib40] MacMillan S, Szeszko PR, Moore GJ, Madden R, Lorch E, Ivey J et al. Increased amygdala: hippocampal volume ratios associated with severity of anxiety in pediatric major depression. J Child Adolesc Psychopharmacol 2003; 13: 65–73. 12804127 10.1089/104454603321666207

[bib41] Rosso IM, Cintron CM, Steingard RJ, Renshaw PF, Young AD, Yurgelun-Todd DA. Amygdala and hippocampus volumes in pediatric major depression. Biol Psychiatry 2005; 57: 21–26. 15607296 10.1016/j.biopsych.2004.10.027

[bib42] Lupien SJ, Parent S, Evans AC, Tremblay RE, Zelazo PD, Corbo V et al. Larger amygdala but no change in hippocampal volume in 10-year-old children exposed to maternal depressive symptomatology since birth. Proc Natl Acad Sci USA 2011; 108: 14324–14329. 21844357 10.1073/pnas.1105371108 PMC3161565

[bib43] Chen MC, Hamilton JP, Gotlib IH. Decreased hippocampal volume in healthy girls at risk of depression. Arch Gen Psychiatry 2010; 67: 270–276. 20194827 10.1001/archgenpsychiatry.2009.202 PMC2845291

[bib44] Jacobs EG, Epel ES, Lin J, Blackburn EH, Rasgon NL. Relationship between leukocyte telomere length, telomerase activity, and hippocampal volume in early aging. JAMA Neurol 2014; 71: 921–923. 25023551 10.1001/jamaneurol.2014.870 PMC5287208

[bib45] Wikgren M, Karlsson T, Lind J, Nilbrink T, Hultdin J, Sleegers K et al. Longer leukocyte telomere length is associated with smaller hippocampal volume among non-demented APOE epsilon3/epsilon3 subjects. PLoS One 2012; 7: e34292. 22506016 10.1371/journal.pone.0034292 PMC3323621

[bib46] Wolkowitz OM, Mellon SH, Lindqvist D, Epel ES, Blackburn EH, Lin J et al. PBMC telomerase activity, but not leukocyte telomere length, correlates with hippocampal volume in major depression. Psychiatry Res 2015; 232: 58–64. 25773002 10.1016/j.pscychresns.2015.01.007 PMC4404215

[bib47] Ferron SR, Marques-Torrejon MA, Mira H, Flores I, Taylor K, Blasco MA et al. Telomere shortening in neural stem cells disrupts neuronal differentiation and neuritogenesis. J Neurosci 2009; 29: 14394–14407. 19923274 10.1523/JNEUROSCI.3836-09.2009 PMC6665809

[bib48] Cheng A, Shin-ya K, Wan R, Tang SC, Miura T, Tang H et al. Telomere protection mechanisms change during neurogenesis and neuronal maturation: newly generated neurons are hypersensitive to telomere and DNA damage. J Neurosci 2007; 27: 3722–3733. 17409236 10.1523/JNEUROSCI.0590-07.2007 PMC6672411

[bib49] Lindqvist D, Mueller S, Mellon SH, Su Y, Epel ES, Reus VI et al. Peripheral antioxidant markers are associated with total hippocampal and CA3/dentate gyrus volume in MDD and healthy controls-preliminary findings. Psychiatry Res 2014; 224: 168–174. 25266915 10.1016/j.pscychresns.2014.09.002 PMC4254356

[bib50] von Zglinicki T. Oxidative stress shortens telomeres. Trends Biochem Sci 2002; 27: 339–344. 12114022 10.1016/s0968-0004(02)02110-2

[bib51] Malykhin NV, Carter R, Seres P, Coupland NJ. Structural changes in the hippocampus in major depressive disorder: contributions of disease and treatment. J Psychiatry Neurosci 2010; 35: 337–343. 20731966 10.1503/jpn.100002 PMC2928287

[bib52] Connolly CG, Wu J, Ho TC, Hoeft F, Wolkowitz O, Eisendrath S et al. Resting-state functional connectivity of subgenual anterior cingulate cortex in depressed adolescents. Biol Psychiatry 2013; 74: 898–907. 23910949 10.1016/j.biopsych.2013.05.036 PMC4103629

[bib53] Ho TC, Connolly CG, Henje Blom E, LeWinn KZ, Strigo IA, Paulus MP et al. Emotion-dependent functional connectivity of the default mode network in adolescent depression. Biol Psychiatry 2014; 78: 635–646. 25483399 10.1016/j.biopsych.2014.09.002 PMC4362932

[bib54] Kaufman J, Birmaher B, Brent D, Rao U, Flynn C, Moreci P et al. Schedule for affective disorders and schizophrenia for school-age children-present and lifetime version (K-SADS-PL): initial reliability and validity data. J Am Acad Child Adolesc Psychiatry 1997; 36: 980–988. 9204677 10.1097/00004583-199707000-00021

[bib55] Poznanski EO. Children's Depression Rating Scale-Revised (CDRS-R) Manual. Western Psychological Services: Los Angeles, 1996, pp 76.

[bib56] Bernstein DP, Ahluvalia T, Pogge D, Handelsman L. Validity of the Childhood Trauma Questionnaire in an adolescent psychiatric population. J Am Acad Child Adolesc Psychiatry 1997; 36: 340–348. 9055514 10.1097/00004583-199703000-00012

[bib57] Dannlowski U, Stuhrmann A, Beutelmann V, Zwanzger P, Lenzen T, Grotegerd D et al. Limbic scars: long-term consequences of childhood maltreatment revealed by functional and structural magnetic resonance imaging. Biol Psychiatry 2012; 71: 286–293. 22112927 10.1016/j.biopsych.2011.10.021

[bib58] Shalev I. Early life stress and telomere length: Investigating the connection and possible mechanisms: a critical survey of the evidence base, research methodology and basic biology. BioEssays 2012; 34: 943–952. 22991129 10.1002/bies.201200084 PMC3557830

[bib59] Cawthon RM. Telomere measurement by quantitative PCR. Nucleic Acids Res 2002; 30: e47. 12000852 10.1093/nar/30.10.e47 PMC115301

[bib60] Gotlib IH, LeMoult J, Colich NL, Foland-Ross LC, Hallmayer J, Joormann J et al. Telomere length and cortisol reactivity in children of depressed mothers. Mol Psychiatry 2014; 20: 615–620. 25266121 10.1038/mp.2014.119 PMC4419149

[bib61] Cox RW. AFNI: software for analysis and visualization of functional magnetic resonance neuroimages. Comput Biomed Res 1996; 29: 162–173. 8812068 10.1006/cbmr.1996.0014

[bib62] Smith SM, Jenkinson M, Woolrich MW, Beckmann CF, Behrens TE, Johansen-Berg H et al. Advances in functional and structural MR image analysis and implementation as FSL. Neuroimage 2004; 23: S208–S219. 15501092 10.1016/j.neuroimage.2004.07.051

[bib63] Zhang Y, Brady M, Smith S. Segmentation of brain MR images through a hidden Markov random field model and the expectation-maximization algorithm. IEEE Trans Med Imaging 2001; 20: 45–57. 11293691 10.1109/42.906424

[bib64] Patenaude B, Smith SM, Kennedy DN, Jenkinson M. A Bayesian model of shape and appearance for subcortical brain segmentation. Neuroimage 2011; 56: 907–922. 21352927 10.1016/j.neuroimage.2011.02.046 PMC3417233

[bib65] R Development Core TeamR: A Language and Environment for Statistical Computing Vol.1). R Foundation for Statistical Computing: Vienna, Austria, 2014.

[bib66] O'Brien LM, Ziegler DA, Deutsch CK, Frazier JA, Herbert MR, Locascio JJ. Statistical adjustments for brain size in volumetric neuroimaging studies: some practical implications in methods. Psychiatry Res 2011; 193: 113–122. 21684724 10.1016/j.pscychresns.2011.01.007 PMC3510982

[bib67] Entringer S, Epel ES, Lin J, Buss C, Shahbaba B, Blackburn EH et al. Maternal psychosocial stress during pregnancy is associated with newborn leukocyte telomere length. Am J Obstet Gynecol 2013; 208: 134.e1–134.e7. 23200710 10.1016/j.ajog.2012.11.033 PMC3612534

[bib68] Stratmann M, Konrad C, Kugel H, Krug A, Schöning S, Ohrmann P et al. Insular and hippocampal gray matter volume reductions in patients with major depressive disorder. PLoS One 2014; 9: e102692. 25051163 10.1371/journal.pone.0102692 PMC4106847

[bib69] Mervaala E, Fohr J, Kononen M, Valkonen-Korhonen M, Vainio P, Partanen K et al. Quantitative MRI of the hippocampus and amygdala in severe depression. Psychol Med 2000; 30: 117–125. 10722182 10.1017/s0033291799001567

[bib70] Vakili K, Pillay SS, Lafer B, Fava M, Renshaw PF, Bonello-Cintron CM et al. Hippocampal volume in primary unipolar major depression: a magnetic resonance imaging study. Biol Psychiatry 2000; 47: 1087–1090. 10862809 10.1016/s0006-3223(99)00296-6

[bib71] Frodl T, Carballedo A, Frey EM, O'Keane V, Skokauskas N, Morris D et al. Expression of glucocorticoid inducible genes is associated with reductions in cornu ammonis and dentate gyrus volumes in patients with major depressive disorder. Dev Psychopathol 2014; 26: 1209–1217. 25422956 10.1017/S0954579414000972

[bib72] Na KS, Chang HS, Won E, Han KM, Choi S, Tae WS et al. Association between glucocorticoid receptor methylation and hippocampal subfields in major depressive disorder. PLoS One 2014; 9: e85425. 24465557 10.1371/journal.pone.0085425 PMC3897456

[bib73] Raz N, Daugherty AM, Bender AR, Dahle CL, Land S. Volume of the hippocampal subfields in healthy adults: differential associations with age and a pro-inflammatory genetic variant. Brain Struct Funct 2014; 220: 2663–2674. 24947882 10.1007/s00429-014-0817-6 PMC4272678

[bib74] Guadalupe T, Zwiers MP, Teumer A, Wittfeld K, Vasquez AA, Hoogman M et al. Measurement and genetics of human subcortical and hippocampal asymmetries in large datasets. Hum Brain Mapp 2014; 35: 3277–3289. 24827550 10.1002/hbm.22401 PMC6869341

[bib75] King KS, Kozlitina J, Rosenberg RN, Peshock RM, McColl RW, Garcia CK. Effect of leukocyte telomere length on total and regional brain volumes in a large population-based cohort. JAMA Neurol 2014; 71: 1247–1254. 25090243 10.1001/jamaneurol.2014.1926 PMC5479062

[bib76] Teicher MH, Anderson CM, Polcari A. Childhood maltreatment is associated with reduced volume in the hippocampal subfields CA3, dentate gyrus, and subiculum. Proc Natl Acad Sci USA 2012; 109: E563–E572. 22331913 10.1073/pnas.1115396109 PMC3295326

[bib77] Huang TT, Zou Y, Corniola R. Oxidative stress and adult neurogenesis—effects of radiation and superoxide dismutase deficiency. Semin Cell Dev Biol 2012; 23: 738–744. 22521481 10.1016/j.semcdb.2012.04.003 PMC3410958

[bib78] Ng F, Berk M, Dean O, Bush AI. Oxidative stress in psychiatric disorders: evidence base and therapeutic implications. Int J Neuropsychopharmacol 2008; 11: 851–876. 18205981 10.1017/S1461145707008401

[bib79] Wolkowitz OM, Mellon SH, Epel ES, Lin J, Reus VI, Rosser R et al. Resting leukocyte telomerase activity is elevated in major depression and predicts treatment response. Mol Psychiatry 2012; 17: 164–172. 21242992 10.1038/mp.2010.133 PMC3130817

[bib80] Gadalla SM, Cawthon R, Giri N, Alter BP, Savage SA. Telomere length in blood, buccal cells, and fibroblasts from patients with inherited bone marrow failure syndromes. Aging 2010; 2: 867–874. 21113082 10.18632/aging.100235 PMC3006028

[bib81] Takubo K, Aida J, Izumiyama-Shimomura N, Ishikawa N, Sawabe M, Kurabayashi R et al. Changes of telomere length with aging. Geriatr Gerontol Int 2010; 10(Suppl 1): S197–S206. 20590834 10.1111/j.1447-0594.2010.00605.x

[bib82] Dlouha D, Maluskova J, Kralova Lesna I, Lanska V, Hubacek JA. Comparison of the relative telomere length measured in leukocytes and eleven different human tissues. Physiol Res 2014; 63(Suppl 3): S343–S350. 25428739 10.33549/physiolres.932856

[bib83] Wei YB, Backlund L, Wegener G, Mathe AA, Lavebratt C. Telomerase dysregulation in the hippocampus of a rat model of depression: normalization by lithium. Int J Neuropsychopharmacol 2015; 18: pyv002. 25618407 10.1093/ijnp/pyv002 PMC4540104

[bib84] Fischl B. FreeSurfer. Neuroimage 2012; 62: 774–781. 22248573 10.1016/j.neuroimage.2012.01.021 PMC3685476

[bib85] Pardoe HR, Pell GS, Abbott DF, Jackson GD. Hippocampal volume assessment in temporal lobe epilepsy: how good is automated segmentation? Epilepsia 2009; 50: 2586–2592. 19682030 10.1111/j.1528-1167.2009.02243.x PMC3053147

[bib86] Wenger E, Martensson J, Noack H, Bodammer NC, Kuhn S, Schaefer S et al. Comparing manual and automatic segmentation of hippocampal volumes: reliability and validity issues in younger and older brains. Hum Brain Mapp 2014; 35: 4236–4248. 24532539 10.1002/hbm.22473 PMC6869097

