# Supplementary material for: Peripheral telomere length and hippocampal volume in adolescents with major depressive disorder
Source: Transl Psychiatry. 2015 Nov 10;5(11):e676–. doi: 10.1038/tp.2015.172 (PMC5068765; doi:10.1038/tp.2015.172)
Supplement: Supplementary Information [file tp2015172x1.docx]

Supplementary material for:

Peripheral Telomere Length and Hippocampal Volume in Adolescents with Major Depressive Disorder

# Supplemental Methods

## Participants

The DSM-IV diagnosis of major depressive disorder (MDD) was validated with the Schedule for Affective Disorders and Schizophrenia for School-Age Children-Present and Lifetime Version ^[1](#_ENREF_1)^. Co-morbidity of anxiety disorders was allowed in the MDD sample. The Diagnostic Interview Schedule for Children Version 4.0 [^2^](#_ENREF_2) and the Diagnostic Predictive Scales [^3^](#_ENREF_3) were used to identify any potential presence of Axis I disorders in the healthy controls (HC). In all participants, depression severity was clinically assessed with the Children’s Depression Rating Scale-Revised (CDRS-R) [^4^](#_ENREF_4) and self-assessed with the Beck Depression Inventory-II (BDI-II) [^5^](#_ENREF_5). Symptoms of anxiety were self-assessed with the Multidimensional Anxiety Scale for Children (MASC) [^6^](#_ENREF_6).

Presence of MRI related contraindications (e.g. pregnancy, metallic implants, claustrophobia), left-handedness, IQ by Wechsler Abbreviated Scale of Intelligence (WASI) [^7^](#_ENREF_7) < 70 and pre-pubertal status self-assed by Tanner stage [^8^](#_ENREF_8), evidence of drug misuse (illicit or prescription), drinking > 2 alcoholic beverages per week or within the month prior to the day of scanning, or the presence of any neurological disorder resulted in exclusion from the study. In addition all participants were clinically assessed using the Children’s Global Assessment Scale (CGAS) [^9^](#_ENREF_9). Participants’ parents also completed a questionnaire for socioeconomic status (Hollingshead Two Factor Index of Social Position, HSP) [^10^](#_ENREF_10).

We excluded participants from the current analysis because of missing or degraded telomere data (n=27). HC subjects that had a CDRS-R score > 54 (n=2) suggesting high levels of depression, as well as MDD subjects with a CDRS-R score < 55 indicative of low depression symptom severity were also excluded (n=2). Several MDD subjects were excluded because of a history of psychotropic medication (n=9). In addition, subjects whose hippocampal volume was above or below 1.5 times the interquartile range of hippocampal volumes in the sample were identified as contributing to non-normal residuals during model testing. These subjects were considered outliers and removed from analysis (n=7). For an overview see Supplemental Table 1.

| Supplemental Table 1. Number of participants excluded from analysis. | | | | |
| --- | --- | --- | --- | --- |
| Reason for exclusion from analysis | Number of exclusions (MDD/HC) | Total exclusions (*n*) | Remaining *n* for analysis |  |
| Missing or degraded TL data | 10/17 | 27 | 137 |  |
| HCs with a CDRS-R score higher than 54 | -/2 | 2 | 135 |  |
| MDDs with a CDRS-R score lower than 55 | 2/- | 2 | 133 |  |
| History of psychotropic medication | 9/- | 9 | 124 |  |
| HV above/below 1.5× IQR of HV | 1/6 | 7 | 117* |  |
| Abbreviations: MDD, major depressive disorder; HC, healthy controls; TL, telomere length;  CDRS-R, Children’s Depression Rating Score – Revised; HV, hippocampal volume;  IQR, interquartile range.  * indicates the final n used for statistical analyses. | | | | |

## Telomere length measurement

The primers for the telomere PCR are *tel1b* [5'-CGGTTT(GTTTGG)_5_GTT-3'], used at a final concentration of 100 nM, and *tel2b* [5'-GGCTTG(CCTTAC)_5_CCT-3'], used at a final concentration of 900 nM. The primers for the single-copy gene (human beta-globin) PCR are *hbg1* [5' GCTTCTGACACAACTGTGTTCACTAGC-3'], used at a final concentration of 300 nM, and *hbg2* [5'-CACCAACTTCATCCACGTTCACC-3'], used at a final concentration of 700 nM. The final reaction mix contains 20 mM Tris-HC, pH 8.4; 50 mM KCl; 200 μM each dNTP; 1% DMSO; 0.4x Syber Green I; 22 ng E. coli DNA per reaction; 0.4 Units of Platinum Taq DNA polymerase (Invitrogen Inc.), 7 ng of genomic DNA per 11 microliter reaction. Tubes containing 26, 8.75, 2.9, 0.97, 0.324 and 0.108ng of a reference DNA (from Hela cancer cells) were included in each PCR run so that the quantity of targeted templates in each research sample could be determined relative to the reference DNA sample by the standard curve method. Identical reference DNA was used for all PCR runs.

To control for inter-assay variability, eight control DNA samples were entered in each run. To obtain a normalizing factor, the T/S ratio of each control DNA was divided by the average T/S for the same DNA from 10 runs in each batch. We completed this procedure for all eight samples and the average normalizing factor for all eight samples was used to correct the participant’s DNA samples, in order to get the final T/S ratio. Subsequently, the T/S ratio for each sample was measured twice. When the duplicate T/S value and the initial value varied by more than 7%, the sample was run the third time and the two values closest to each other were reported. By adopting this method, the average CV for this study was 2.1%.

# Supplemental Results

## Between-group analyses without covariates

An additional ANCOVA tested for the between-group difference in TL without adjusting for age and sex. It showed that the depressed group still exhibited significantly shorter TL compared to the HC (F_(1,115)_ = 6.43, *p* = 0.013). Similarly, the TBV-adjusted between-group difference in the right hippocampal volume (HV) remained significant when not controlling for age and sex (F_(1,114)_ = 6.67, *p* = 0.011), with MDDs showing lower right HV. Consistent with the model in the main manuscript, no between-group difference in the left HV was observed (F_(,1,114)_ = 0.23, *p* = 0.64) when controlling for TBV, but not for age and sex. In summary, controlling for age and sex in the between-group comparison of both TL and HV (left, right) did not significantly alter the results.

## Telomere length and demographic and clinical variables

TL was significantly associated with age across groups (β = -0.037, t_(114)_ = -2.21, *p <* 0.05), but not within each group separately (both *p* > 0.095).TL was not significantly associated with MASC in either group (all *p* > 0.5). Associations across groups were adjusted for diagnostic group.

## Hippocampal volume and demographic and clinical variables

Left hippocampal volume was significantly associated with age within MDDs (β = 68.3, t_(51)_=2.14, *p <* 0.05), but not within HCs (*p* = 0.29) or across diagnostic group (*p =* 0.06), whereas the right HV was significantly associated with age across diagnostic group (β = 54.6, t_(113)_ = 2.44, *p <* 0.05), but only marginally so within each group (both *p >* 0.07). Furthermore, HV was not associated with MASC scores in either group (all *p >* 0.63). All associations were adjusted for total brain volume and across group regressions were adjusted for diagnostic group.

## Interaction models

An additional model specifically tested for the interaction of group and age on TL while adjusting for sex, but yielded no significant interaction (F_(1,,112)_= 0.01, *p* = 0.92). Likewise, the interaction of group and sex on TL also did not reveal a significant effect (F_(1, 112)_ = 0.07, *p* = 0.79). In the same fashion, further analysis testing for interactions on the left and right HV while adjusting for sex and TBV also did not yield significant interaction effects of group and age (left: F_(1, 111)_= 0.008, *p* = 0.93), and (right: F_(1,111)_= 0.004, *p* = 0.95). Finally, there was also no group and sex interaction on the left (F_(1, ,111)_= 0.87, *p* = 0.35) or right HV (F_(1,,111)_= 0.03, *p* = 0.85), after adjusting for age and TBV. We therefore opted to report the most parsimonious models in the manuscript, testing only for the main effects of group, age, and sex on TL and HV (left, right).

## Laterality analysis

To test whether the HV reduction was lateralized we computed an additional ANCOVA with HV as the dependent variable, group (MDD, HC) as the between-subjects factor, laterality (left vs. right) as a within-subjects factor, and age, sex, and total brain volume as covariates. Consistent with the model excluding laterality in the main manuscript, a main effect of group was observed, F_(1,228)_ = 4.42, *p* = 0.036, indicating smaller HV in the depressed group compared to HC after adjusting for laterality, age, sex, and TBV. In addition, a main effect of laterality occurred, F_(1, 228)_ = 4.07, *p* = 0.045, showing larger HV in the right hemisphere, after controlling for diagnostic group, age, sex, and TBV.

## Comorbidity analysis

Comorbid psychiatric disorders assessed during the KSAD-PL interview, a diagnostic interview that MDD participants had to undergo to confirm a primary diagnosis of MDD, and, therefore, eligibility for the current study. We investigated the main effect of the number of comorbidities on both telomere length and hippocampal volume (left, right) by conducting an analysis of variance with number of comorbidities (0, 1, 2+) as a between-subjects factor (and total brain volume as a within-subject factor in case of the hippocampal volume analyses). There was no significant main effect of number of comorbidities on TL, see Supplemental Table 2. In addition, TL values across the number of comorbidities are graphically displayed in Supplemental Figure 1. There was also no main effect of comorbidity on the left or right HV, after adjusting for TBV; see Supplemental Table 2 and Supplemental Figure 1. Twenty subjects had no comorbid psychiatric disorders, 17 subjects had one other psychiatric comorbidity, 12 subjects had two comorbidities, and one subject had five comorbid psychiatric disorders and was therefore binned under two or more comorbidities. Four subjects were missing comorbidity data and were therefore excluded from analyses.

**Supplemental Table 2.** Telomere length and hippocampal volume (left and right) across the number of comorbidities. Values shown are mean ± SD of the TL (T/S Ratio) or hippocampal volume (μL).

|  | **No comorbidities** | **1 comorbidity** | **2+ comorbidities** | **Statistic** |
| --- | --- | --- | --- | --- |
| **TL** | 1.33 ± 0.24 | 1.42 ± 0.25 | 1.43 ± 0.25 | F_(2,47)_ = 0.88, *p* = 0.42 |
| **Left HV** | 3915 ± 380 | 3815 ± 464 | 3799 ± 386 | F_(2,46)_ = 0.75, *p* = 0.48 |
| **Right HV** | 3921 ± 334 | 3854 ± 571 | 3792 ± 373 | F_(2,46)_ = 0.60, *p* = 0.56 |

Abbreviations: TL, telomere length; HV, hippocampal volume. Four subjects were missing comorbidity data and were excluded from analyses. Hippocampal statistics were adjusted for total brain volume.

## Power calculation

To determine the minimum number of participants needed for our study, we derived effect sizes (Cohen’s d) from a number of number of recent articles comparing telomere length and hippocampal volume between depressed, at-risk of depression, and healthy individuals (also referred to in the main manuscript [^11-15^](#_ENREF_11)). The average effect size for between-group differences in these studies was d=0.589. Assuming a desired statistical power level of 0.8 and p=0.05, the minimum total number of participants for two-tailed tests is 94 (47 per group) to detect significant differences. Since the number of participants in our sample consists of 54 MDD and 63 well-matched healthy controls, these calculations suggest that our study is sufficiently powered to detect significant differences.


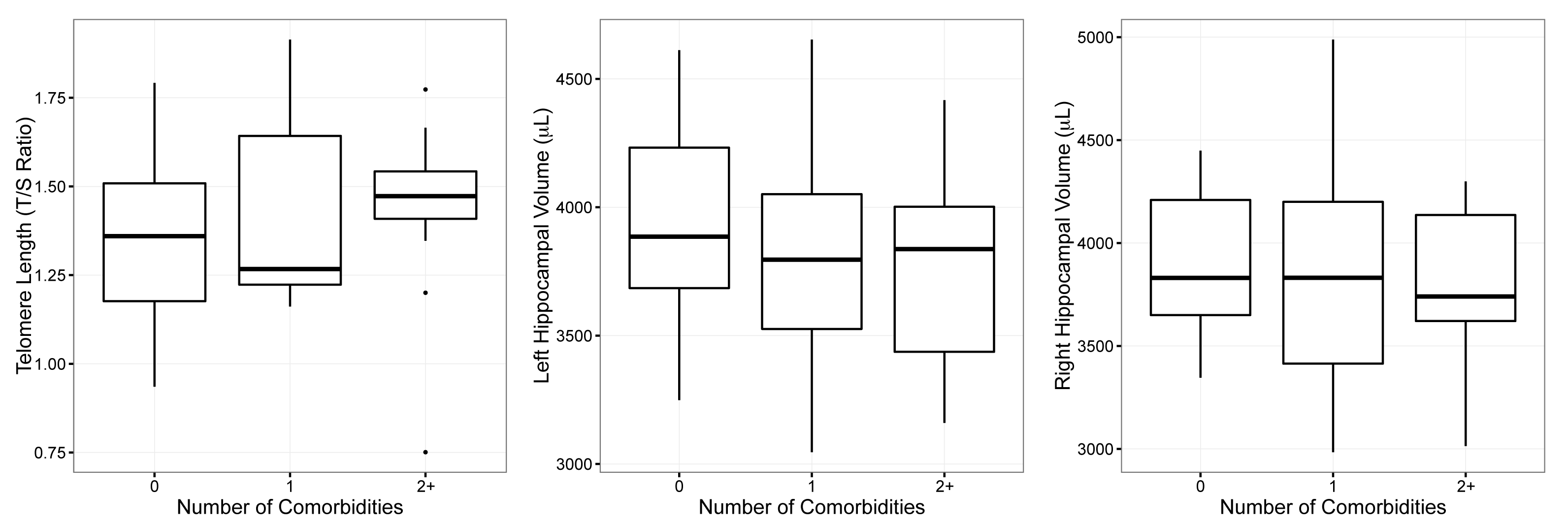


**Supplemental Figure 1.** Telomere Length (T/S Ratio) and hippocampal volume (μL) as a function of the number of comorbid psychiatric disorders in adolescents with major depressive disorder.

## Check for homogeneity of variance

All data was visually inspected using boxplots and subjected to Levene's test for homogeneity of variance. No data subjected to ANCOVA violated the assumption of homoscedasticity. None of the t-tests reported in Table 1 in the manuscript assumed equality of variance between the samples, as we used the Welch approximation to the degrees of freedom.

# Supplemental Discussion

When assessing HV in adolescents it should also be taken into consideration that the structural development of the hippocampus is heterogeneous, so that posterior sub-regions increase in volume over time whereas anterior regions decrease [^16^](#_ENREF_16). Importantly, the normal developmental trajectory of the hippocampus also shows a nonlinear increase in gray matter HV over time, with peak volume in pre-adolescence[^17^](#_ENREF_17)^,^ [^18^](#_ENREF_18). Even though these developmental aspects of the HV complicate the assessment of hippocampus volume in depressed teenagers [^16^](#_ENREF_16), the age-range in our sample is relatively narrow (13-18 years old) and HV growth is assumed to be linear over this range. Furthermore, puberty plays a role in normal hippocampus development. Pre-pubertal males and females have similar hippocampal volumes, whereas post-pubertal females have significantly larger bilateral hippocampi [^19^](#_ENREF_19) and it has been shown that hippocampal volumes seem to vary as a function of pubertal status regardless of sex, and were associated with circulating testosterone levels [^20^](#_ENREF_20). In the present study all subjects were post-pubertal but testosterone levels were not assessed. Sex differences of HV have also been reported in adult MDD; with greater HV loss in males [^21^](#_ENREF_21)^,^ [^22^](#_ENREF_22). No such differences were found in our study.

The Childhood Trauma Questionnaire (CTQ) has been validated in many studies of similar populations, but there are concerns that retrospective reports of trauma are biased by one's current emotional state [^23^](#_ENREF_23). A more complete childhood trauma assessment, ideally prospective and longitudinal, could have provided important information to our study for the following reasons; first, the prevalence of childhood maltreatment is highly elevated in populations suffering from MDD [^24^](#_ENREF_24), second, childhood maltreatment or abuse, and even prenatal exposure to maternal stress have been associated with shorter TL in children and adults, for a review, see [^25^](#_ENREF_25) and it has been suggested that telomere erosion is a potential mechanism linking childhood stress to health problems such as depression later in life [^26^](#_ENREF_26)^,^ [^27^](#_ENREF_27); third, It has been hypothesized that exposure to early adversity may induce a series of events leading to an attenuation of the typical pattern of hippocampal growth across adolescence, in turn predisposing for depressive illness [^28^](#_ENREF_28)^,^ [^29^](#_ENREF_29). The described reductions of TL and hippocampal volumes in the MDD group could thus be the result of periods of repeated neurotoxic stress accumulated during stress related to early adverse life events [^30^](#_ENREF_30) rather than to MDD itself, which our data does not support.

# References

1. Kaufman J, Birmaher B, Brent D, Rao U, Flynn C, Moreci P *et al.* Schedule for Affective Disorders and Schizophrenia for School-Age Children-Present and Lifetime Version (K-SADS-PL): initial reliability and validity data. *J Am Acad Child Adolesc Psychiatry* 1997; **36**(7)**:** 980-988.

2. Shaffer D, Fisher P, Lucas CP, Dulcan MK, Schwab-Stone ME. NIMH Diagnostic Interview Schedule for Children Version IV (NIMH DISC-IV): description, differences from previous versions, and reliability of some common diagnoses. *J Am Acad Child Adolesc Psychiatry* 2000; **39**(1)**:** 28-38.

3. Lucas CP, Zhang H, Fisher PW, Shaffer D, Regier DA, Narrow WE *et al.* The DISC Predictive Scales (DPS): efficiently screening for diagnoses. *J Am Acad Child Adolesc Psychiatry* 2001; **40**(4)**:** 443-449.

4. Poznanski EO. *Children's Depression Rating Scale-Revised (CDRS-R) Manual*. Western Psychological Services, Los Angeles1996, 76pp.

5. Beck AT, Steer RA, Ball R, Ranieri W. Comparison of Beck Depression Inventories -IA and -II in psychiatric outpatients. *Journal of personality assessment* 1996; **67**(3)**:** 588-597.

6. March JS, Parker JD, Sullivan K, Stallings P, Conners CK. The Multidimensional Anxiety Scale for Children (MASC): factor structure, reliability, and validity. *J Am Acad Child Adolesc Psychiatry* 1997; **36**(4)**:** 554-565.

7. Wechsler D. *Wechsler Abbreviated Scale of Intelligence*. Harcourt Brace & Company: New York, 1999.

8. Tanner JM. The Regulation of Human Growth. *Child development* 1963; **34:** 817-847.

9. Dyrborg J, Larsen FW, Nielsen S, Byman J, Nielsen BB, Gautre-Delay F. The Children's Global Assessment Scale (CGAS) and Global Assessment of Psychosocial Disability (GAPD) in clinical practice--substance and reliability as judged by intraclass correlations. *Eur Child Adolesc Psychiatry* 2000; **9**(3)**:** 195-201.

10. Hollingshead AB. *Two Factor Index of Social Position*. Mimeo: Yale University, New Haven, Connecticut, 1957.

11. Simon NM, Smoller JW, McNamara KL, Maser RS, Zalta AK, Pollack MH *et al.* Telomere shortening and mood disorders: preliminary support for a chronic stress model of accelerated aging. *Biol Psychiatry* 2006; **60**(5)**:** 432-435.

12. Chen MC, Hamilton JP, Gotlib IH. Decreased hippocampal volume in healthy girls at risk of depression. *Arch Gen Psychiatry* 2010; **67**(3)**:** 270-276.

13. Rao U, Chen LA, Bidesi AS, Shad MU, Thomas MA, Hammen CL. Hippocampal changes associated with early-life adversity and vulnerability to depression. *Biol Psychiatry* 2010; **67**(4)**:** 357-364.

14. MacMaster FP, Mirza Y, Szeszko PR, Kmiecik LE, Easter PC, Taormina SP *et al.* Amygdala and hippocampal volumes in familial early onset major depressive disorder. *Biol Psychiatry* 2008; **63**(4)**:** 385-390.

15. Caetano SC, Fonseca M, Hatch JP, Olvera RL, Nicoletti M, Hunter K *et al.* Medial temporal lobe abnormalities in pediatric unipolar depression. *Neurosci Lett* 2007; **427**(3)**:** 142-147.

16. Gogtay N, Nugent TF, 3rd, Herman DH, Ordonez A, Greenstein D, Hayashi KM *et al.* Dynamic mapping of normal human hippocampal development. *Hippocampus* 2006; **16**(8)**:** 664-672.

17. Ostby Y, Tamnes CK, Fjell AM, Westlye LT, Due-Tonnessen P, Walhovd KB. Heterogeneity in subcortical brain development: A structural magnetic resonance imaging study of brain maturation from 8 to 30 years. *J Neurosci* 2009; **29**(38)**:** 11772-11782.

18. Uematsu A, Matsui M, Tanaka C, Takahashi T, Noguchi K, Suzuki M *et al.* Developmental trajectories of amygdala and hippocampus from infancy to early adulthood in healthy individuals. *PLoS One* 2012; **7**(10)**:** e46970.

19. Satterthwaite TD, Vandekar S, Wolf DH, Ruparel K, Roalf DR, Jackson C *et al.* Sex differences in the effect of puberty on hippocampal morphology. *J Am Acad Child Adolesc Psychiatry* 2014; **53**(3)**:** 341-350 e341.

20. Neufang S, Specht K, Hausmann M, Gunturkun O, Herpertz-Dahlmann B, Fink GR *et al.* Sex differences and the impact of steroid hormones on the developing human brain. *Cerebral cortex (New York, NY : 1991)* 2009; **19**(2)**:** 464-473.

21. Frodl T, Meisenzahl EM, Zetzsche T, Born C, Groll C, Jager M *et al.* Hippocampal changes in patients with a first episode of major depression. *Am J Psychiatry* 2002; **159**(7)**:** 1112-1118.

22. Kronmuller KT, Pantel J, Gotz B, Kohler S, Victor D, Mundt C *et al.* Life events and hippocampal volume in first-episode major depression. *J Affect Disord* 2008; **110**(3)**:** 241-247.

23. Liu X, Li L, Xiao J, Yang J, Jiang X. Abnormalities of autobiographical memory of patients with depressive disorders: a meta-analysis. *Psychology and psychotherapy* 2013; **86**(4)**:** 353-373.

24. Opel N, Redlich R, Zwanzger P, Grotegerd D, Arolt V, Heindel W *et al.* Hippocampal atrophy in major depression: a function of childhood maltreatment rather than diagnosis? *Neuropsychopharmacology* 2014; **39**(12)**:** 2723-2731.

25. Shalev I, Entringer S, Wadhwa PD, Wolkowitz OM, Puterman E, Lin J *et al.* Stress and telomere biology: A lifespan perspective. *Psychoneuroendocrinology* 2013; **38**(9)**:** 1835-1842.

26. Shalev I. Early life stress and telomere length: Investigating the connection and possible mechanisms: A critical survey of the evidence base, research methodology and basic biology. *BioEssays : news and reviews in molecular, cellular and developmental biology* 2012; **34**(11)**:** 943-952.

27. Teicher MH, Anderson CM, Polcari A. Childhood maltreatment is associated with reduced volume in the hippocampal subfields CA3, dentate gyrus, and subiculum. *Proc Natl Acad Sci U S A* 2012; **109**(9)**:** E563-572.

28. Andersen SL, Teicher MH. Stress, sensitive periods and maturational events in adolescent depression. *Trends Neurosci* 2008; **31**(4)**:** 183-191.

29. Dannlowski U, Stuhrmann A, Beutelmann V, Zwanzger P, Lenzen T, Grotegerd D *et al.* Limbic scars: long-term consequences of childhood maltreatment revealed by functional and structural magnetic resonance imaging. *Biol Psychiatry* 2012; **71**(4)**:** 286-293.

30. Cheng YQ, Xu J, Chai P, Li HJ, Luo CR, Yang T *et al.* Brain volume alteration and the correlations with the clinical characteristics in drug-naive first-episode MDD patients: a voxel-based morphometry study. *Neurosci Lett* 2010; **480**(1)**:** 30-34.
